# Upregulation of miR-34a by Inhibition of IRE1*α* Has Protective Effect against A*β*-Induced Injury in SH-SY5Y Cells by Targeting Caspase-2

**DOI:** 10.1155/2019/2140427

**Published:** 2019-06-02

**Authors:** Qianqian Li, Tingjiao Liu, Shanshan Yang, Zhongling Zhang

**Affiliations:** Department of Neurology, The First Affiliated Hospital of Harbin Medical University, Harbin, China

## Abstract

**Background:**

Neurotoxicity induced by the amyloid-*β* (A*β*) peptide is one of the most important pathological mechanisms of Alzheimer's disease (AD). Based on accumulating evidence in AD research, both endoplasmic reticulum stress (ER stress) and alterations in the microRNA (miRNA) network contribute to the pathogenesis of the disease, making them potential therapeutic targets for AD. The present study was performed to investigate whether miR-34a and the inositol-requiring enzyme 1 (IRE1) are involved in the regulation of A*β*-induced cytotoxicity.

**Methods:**

Human neuroblastoma SH-SY5Y cells were treated with A*β*1-40. Cell viability was assessed by the MTT assay. The integrity of the plasma membrane was assessed by LDH release. The expression levels of XBP1s, IRE1*α*, p-IRE1*α*, and Caspase-2 were detected by Western blot analysis. Spliced-XBP1 mRNA and miR-34a were detected by reverse transcription- (RT-) PCR and quantitative real-time PCR, respectively. Caspase-2 activity was measured using the Caspase-2 cellular activity assay kit. The IRE1 inhibitor (STF-083010) was used to determine the role of IRE1*α* on miR-34a expression. SH-SY5Y cells were transfected with miR-34a mimics to assess the role of miR-34a on the activation of Caspase-2 and the viability of A*β*-exposed SH-SY5Y cells.

**Results:**

We showed that A*β* caused concentration- and duration-dependent death of SH-SY5Y cells. The expression levels of XBP1s, p-IRE1*α*, and Caspase-2 were increased, along with a corresponding decrease in the miR-34a levels in A*β*-exposed SH-SY5Y cells. The IRE1 inhibitor (STF-083010) upregulated the expression of miR-34a and suppressed the activation of Caspase-2, effectively alleviating the A*β*-induced death of SH-SY5Y cells. Transfection studies show that miR-34a mimics inhibit the expression of Caspase-2 and restore the viability of A*β*-exposed SH-SY5Y cells.

**Conclusion:**

A*β* peptide induced downregulation of miR-34a through the activation of IRE1*α*, which may induce cytotoxicity by targeting Caspase-2. Upregulation of miR-34a by inhibition of IRE1*α* has protective effects against A*β*-induced injury in SH-SY5Y cells.

## 1. Introduction

Alzheimer's disease (AD) is a chronic and progressive neurodegenerative disease of the central nervous system. It is characterized by extracellular aggregates of amyloid-*β* (A*β*) peptide in senile plaques [1]. Previous studies provide strong evidence that A*β* plays a central role in the pathogenesis of AD [2–4].

The endoplasmic reticulum (ER) is responsible for controlling proper protein synthesis, posttranslational modifications, and protein folding, which is crucial for cell survival. ER stress occurs when the accumulation of nonfolded or misfolded proteins exceeds the processing capacity of the ER under physiological conditions [5]. When cells fail to recover from ER stress, the unfolded protein response (UPR) triggers apoptosis [6]. Inositol-requiring enzyme 1 (IRE1), a transmembrane protein that has both Ser/Thr protein kinases and endoribonuclease activities, is one of the major ER sensors that responds to the aggregation of unfolded proteins [7, 8]. Two types of IRE1 are expressed in mammals, IRE1*α* and IRE1*β*. IRE1*α* is widely expressed in a variety of cells, whereas IRE1*β* is only expressed in the intestine and lung. Activation of IRE1*α* involves its dimerization, oligomerization, and autophosphorylation. Upon activation, IRE1*α* excises a 26-base intron from the x-box binding protein 1 (XBP1) mRNA, producing an active transcription factor (XBP1s) [9–12]. IRE1*α* and XBP1s are key molecules regulating ER stress; the activation of IRE1*α* and the upregulation of the XBP1s expression are often used as markers of the UPR to induce ER stress [13]. However, IRE1*α* acts as a double-edged sword. If restoring ER homeostasis fails, IRE1*α* initiates apoptosis by regulating a large list of substrates, which may eventually induce cell death [14, 15].

MicroRNAs (miRNAs), a type of small noncoding mRNA containing approximately 22 nucleotides, regulate the expression of at least 30% of all genes through binding of the 3′-untranslated region of their target mRNAs, leading to their inhibition of translation or destabilization [16–18]. miR-34a, a gene product of chromosomal locus 1p36.23, is particularly pronounced in the central nervous system (CNS) [19]. Increasing evidence shows that miR-34a dysfunction may contribute to pathologies of the CNS [20, 21]. IRE1*α* has recently been shown to cleave miR-34a, resulting in activation of Caspase-2, which may help initiate apoptosis [22, 23]. Based on previous studies, we explored whether there is activated IRE1*α* and abnormal expression of miR-34a in A*β*-treated SH-SY5Y cells to clarify the role of the IRE1*α*/miR-34a pathway in A*β*-induced cytotoxicity.

## 2. Materials and Methods

### 2.1. Reagents

The A*β*1-40 peptide was obtained from Sigma-Aldrich (St. Louis, MO, USA). STF-083010, a specific IRE1*α* I endonuclease inhibitor, was bought from Sigma-Aldrich and was prepared fresh in a dark room with DMSO for a 25 mM stock solution. The RNA Fast Isolation kit and M-MLV Reverse Transcriptase were obtained from BioTeke Corporation (Beijing, China). The SYBR Green PCR Kit was purchased from Solarbio Science & Technology (Beijing, China), and the Caspase-2 cellular activity assay kit was purchased from the Nanjing Jiancheng Bioengineering Institute (Nanjing, China). The LDH Activity Assay Kit was purchased from Abcam. The miR-34a mimic oligonucleotide and control RNA were purchased from GenePharma (Shanghai, China). The Lipofectamine™ 2000 reagent was obtained from Invitrogen Life Technologies (Carlsbad, CA, USA). Antibodies for the Western blot assay were purchased as follows: XBP1, IRE1*α*, and p-IRE1*α* antibodies (Abcam, Cambridge, UK) and Caspase-2 and *β*-actin antibodies (Sigma-Aldrich, St. Louis, MO, USA). All culture media and fetal bovine serum (FBS) were obtained from Gibco (Carlsbad, CA, USA). Unless specifically mentioned, all other reagents were purchased from Sigma-Aldrich.

### 2.2. A*β* Peptide Preparation

A*β*1-40 was dissolved in double deionized water at a concentration of 200 *μ*mol/l and was stored at −80°C. To increase the toxicity, it was incubated at 37°C for 24 h before use in each of the experiments according to our previous study [24], after which it was diluted to the required concentration.

### 2.3. Cell Culture and Treatment

Human neuroblastoma SH-SY5Y cells were purchased from the Institute of Basic Medical Sciences of the Chinese Academy of Medical Sciences and were cultured in RPMI 1640 medium supplemented with 10% FBS, 300 *μ*g/ml glutamine, 100 *μ*g/ml streptomycin, and 100 U/ml penicillin. Cells were maintained at 37°C in an incubator containing 5% CO_2_. When grown to 90% confluence, cells were washed with serum-free medium and then treated with various concentrations of A*β*1-40 (0, 1, 3, or 5 *μ*M) for 0–48 h. In the inhibition studies, cells were pretreated with STF-083010 (10, 30, 60, or 100 *μ*M) for 6 h, followed by exposure to 5 *μ*M A*β*1-40 for another 24 h [25]. Cell morphology was observed with a phase contrast microscope (AE31; Motic, China).

### 2.4. Transfection

SH-SY5Y cells (1 × 10^5^) were seeded into 6-well plates and cultured for 24 h. On the following day, cells were transfected with 100 nM of miR-34a mimic oligonucleotide or a nonspecific control RNA using the Lipofectamine™ 2000 reagent, according to the manufacturer's instructions. The following sequences were used: miR-34a mimic oligonucleotide 5′-UGGCAGUGUCUUAGCUGGUUGU-3′ and negative control 5′-UCCUAACCGCGUGUCACGUTT-3′. Specifically, serum-free MEM medium (250 *μ*l) was used to dilute the miR-34a mimic (10 *μ*l) and NC. Then, 5 *μ*l of Lipofectamine 2000 was diluted with 250 *μ*l of the same medium and incubated at room temperature for 5 min. The diluted miR-34a mimic or NC was then mixed with the diluted Lipofectamine 2000 and incubated for 20 min at room temperature to allow for complex formation. The complexes were then added to each well containing the SH-SY5Y cells and placed in a 37°C/5% CO_2_ incubator. After 6 h, the cultures were replaced with 2 ml fresh medium supplemented with 10% FBS. Forty-eight hours after transfection, cells were incubated using 5 *μ*M A*β*1-40 for 24 h.

### 2.5. Cytotoxicity Assessment

The viability of SH-SY5Y cells was measured by the 3-(4,5-dimethylthiazol-2-yl)-2,5-diphenyltetrazolium bromide (MTT) assay. SH-SY5Y cells were seeded into 96-well plates at a density of 5 × 10^4^ cells per well and cultured at 37°C in culture medium for 24 h. Cells were washed in sodium medium, 20 *μ*l MTT (5 mg/ml) was added to the cells, and they were cultured at 37°C for 4 h before the addition of stop solution, which was added to dissolve MTT. The absorbance of samples was measured at 490 nm with a microplate reader. Data are presented as the percentage of survival relative to normal cultured cells without any treatment.

The integrity of the plasma membrane was evaluated by determining the release of the cytoplasmic enzyme LDH. The cells were subjected to different treatments. Subsequently, the activity of released LDH was examined according to the instructions of the LDH Activity Assay Kit. Absorbance was measured at a wavelength of 490 nm. The LDH release was expressed as a percentage relative to the positive control group.

### 2.6. Western Blot Assay

After drug treatment, SH-SY5Y cells were harvested and total protein was isolated. The protein concentration was determined by BCA assay. Equal amounts of protein were resolved in 10% SDS-PAGE followed by transfer to a nitrocellulose membrane. Membranes were blocked with 5% fat-free milk in TBST and then immunoblotted with an anti-p-IRE1*α* antibody (diluted 1 : 1000), anti-IRE1*α* antibody (diluted 1 : 1000), anti-XBP1 antibody (diluted 1 : 1000), anti-Caspase-2 antibody (diluted 1 : 1000), or anti-actin antibody (diluted 1 : 1000) at room temperature for 1.5 h. Membranes were washed and incubated with peroxidase-conjugated secondary antibodies at room temperature for 1 h. Subsequently, membranes were exposed to chemiluminescence reagents. Protein expression was quantified using the AlphaEase FC image analysis software.

### 2.7. Reverse Transcription Polymerase Chain Reaction (RT-PCR)

Reverse transcription-PCR was performed to examine the expression of spliced-XBP1 mRNA. Briefly, the total RNA was extracted using the RNApure Total RNA Fast Isolation Kit. Equal amounts of RNA were used for reverse transcription with M-MLV reverse transcriptase, according to the manufacturer's instructions. The cDNAs were then used for PCR amplification. The primers used are listed in Table 1. The following PCR program was used: 95°C for 5 min, followed by 36 cycles of 20 sec at 95°C, 20 sec at 52°C, and 30 sec at 72°C. *β*-Actin served as the internal control for data normalization. PCR products were separated on acrylamide gels containing 7.5% acrylamide for XBP1 PCR products. For quantitative analysis, intensities of DNA bands were quantified using software.

### 2.8. Quantitative Real-Time Polymerase Chain Reaction (qRT-PCR)

The expression level of miR-34a was determined by qRT-PCR. Briefly, total RNA was isolated from cells using the RNApure Total RNA Fast Isolation Kit and was then reverse-transcribed using the Power RT Kit. Real-time PCR was performed using 2x Power Taq PCR MasterMix and SYBR Green on a Superior 5-color Real-Time Quantitative PCR system (Bioneer, Korea). The primers for mature miR-34a and U6 were purchased from Sangon Biotech (China). The primers used are listed in Table 2. The relative amount of miR-34a was normalized to the U6 snRNA, and the fold change in each miRNA was calculated using the 2^-ΔΔCt^ method [26].

### 2.9. Measurement of Caspase-2 Activity in SH-SY5Y Cells

Caspase-2 activity was measured in the lysates of SH-SY5Y cells using the Caspase-2 cellular activity assay kit. Briefly, SH-SY5Y cells were cultured as described above. After washing and trypsinization, cell suspensions were centrifuged (400 g, 3 min, 4°C), and cell pellets were resuspended in 100 *μ*l of ice-cold cell lysis buffer and incubated for 15 min. Following centrifugation (16,000 g, 15 min, 4°C), supernatants were collected, and enzyme activity was measured according to the manufacturer's instructions. The reaction mixtures (total volume 100 *μ*l) were incubated at 37°C overnight, and the optical density value was measured at 405 nm using a microplate reader.

### 2.10. Statistical Analysis

Data are presented as means ± standard errors of the means (SEM). Each procedure was performed in four independent experiments. Statistical analyses were performed using a one-way analysis of variance (ANOVA), followed by an LSD post hoc test. A *P* value < 0.05 was considered significant. All statistical analyses were performed using SPSS software version 13.0.

## 3. Results

### 3.1. A*β*1-40 Induced Cytotoxicity of SH-SY5Y Cells

**Figure 1:**
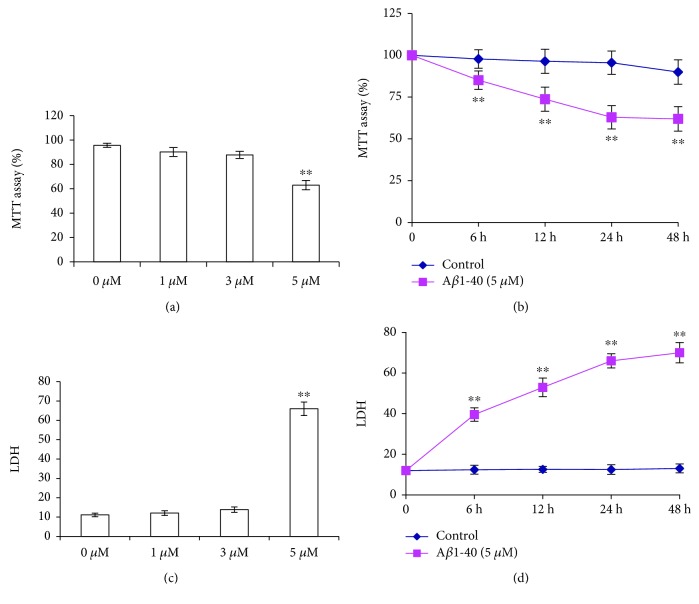
A*β*-induced toxicity in cultured SH-SY5Y cells. Cells were treated with 0, 1, 3, or 5 *μ*M A*β*1-40 for 24 h or treated with 5 *μ*M A*β* for 0, 6, 12, 24, or 48 h. Cell viability was measured using the MTT assay. The integrity of the plasma membrane was assessed by LDH release. Values are presented as the means ± SEM from four independent experiments. ^∗∗^*P* < 0.01 vs. control.

We examined the effect of A*β* on the cytotoxicity of SH-SY5Y cells by MTT and LDH assay. A*β*1-40 induced a concentration- and duration-dependent decrease in the viability of SH-SY5Y cells. Exposure to 1, 3, or 5 *μ*M of A*β*1-40 reduced the survival of SH-SY5Y cells to 90.12 ± 3.68%, 87.73 ± 2.93%, and 62.93 ± 3.83% of the control, respectively (Figure 1(a), *P* < 0.01). We used LDH to further determine the cell viability. Similar to that observed in the MTT assay, A*β*1-40 treatment increased LDH release from 11.11 ± 0.95% to 11.24 ± 0.79%, 12.75 ± 0.90%, and 65.27 ± 0.72% (Figure 1(c), *P* < 0.01). Exposure to 5 *μ*M A*β*1-40 resulted in sufficient toxicity in the cells and was used in subsequent studies. Exposure to 5 *μ*M of A*β*1-40 for 6, 12, 24, or 48 h reduced the survival of SH-SY5Y cells to 85.12 ± 5.54%, 73.75 ± 7.22%, 62.93 ± 6.99%, and 61.95 ± 7.32% of that of the control, respectively (Figure 1(b), *P* < 0.01). Exposure to 5 *μ*M of A*β*1-40 for 6, 12, 24, or 48 h increased the LDH release to 37.81 ± 0.77%, 50.76 ± 0.62%, 65.27 ± 0.72%, or 68.54 ± 2.20%, respectively (Figure 1(d), *P* < 0.01).

### 3.2. Activation of the IRE1 Signaling Pathway in A*β*-Treated SH-SY5Y Cells

**Figure 2:**
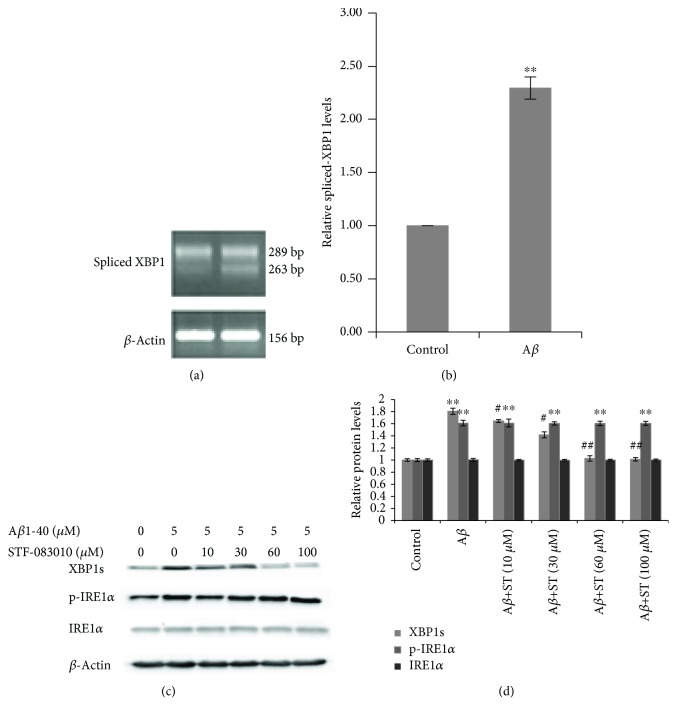
Activation of the IRE1 signaling pathway in A*β*-treated SH-SY5Y cells. SH-SY5Y cells were incubated with 5 *μ*M A*β*1-40 for 24 h. The spliced-XBP1 level was detected by RT-PCR. (a) Representative RT-PCR data showing the spliced-XBP1 bands. (b) Densitometric analysis of the spliced-XBP1 level normalized to the *β*-actin level. The cultured SH-SY5Y cells were pretreated with STF-083010 (0, 10, 30, 60, or 100 *μ*M) for 6 h, followed by exposure to 5 *μ*M A*β*1-40 under the continued presence of STF-083010 for 24 h. The levels of XBP1s, p-IRE1*α*, and IRE1*α* were detected by Western blotting. (c) Representative Western blot showing the XBP1s, p-IRE1*α*, and IRE1*α* bands. (d) Densitometric analysis of the protein levels of XBP1s, p-IRE1*α*, and IRE1*α* normalized to the *β*-actin level. The values are presented as the means ± SEM from four independent experiments. ^∗∗^*P* < 0.01 compared with the control. ^#^*P* < 0.05 compared with the A*β*1-40-alone group. ^##^*P* < 0.01 compared with the A*β*1-40-alone group.

The IRE1 signaling pathway through XBP1 is strongly linked with ER stress and UPR. To investigate whether IRE1*α*-mediated UPR post-A*β*1-40 injury is activated, spliced-XBP1 mRNA was measured by RT-PCR. The expression of IRE1*α*, phosphorylated IRE1*α* (p-IRE1*α*), and XBP1s in SH-SY5Y cells was measured by Western blotting. Exposure to A*β*1-40 (5 *μ*M) for 24 h increased the level of the spliced-XBP1 mRNA in SH-SY5Y cells (2.29-fold vs. control, *P* < 0.01) (Figures 2(a) and 2(b)). Increased expression of the p-IRE1*α* (1.61-fold vs. control, *P* < 0.01) (Figures 2(c) and 2(d)) and spliced-XBP1 (XBP1s) proteins was also observed in A*β*-treated SH-SY5Y cells (1.80-fold vs. control, *P* < 0.01) (Figures 2(c) and 2(d)). STF-083010, an inhibitor of IRE1's RNase, was applied to prevent the splicing of XBP1. We found that STF-083010 inhibits IRE1*α* in a dose-dependent manner and the effect was reflected by XBP1 splicing. XBP1 splicing was reduced to normal levels when treated with STF-083010 at 60 *μ*M or above (*P* < 0.01, Figures 2(c) and 2(d)). Total IRE1*α* remained unchanged post-A*β*1-40 injury and STF-081030 treatment. At the same time, STF-081030 treatment could not alleviate the overexpression of p-IRE1*α* after A*β*1-40 injury. These results suggest that STF-083010 blocks IRE1-dependent splicing of XBP1 but not IRE1-autophosphorylation in response to A*β* (1-40) (Figures 2(c) and 2(d)).

### 3.3. IRE1*α* Inhibition Upregulated miR-34a Expression

**Figure 3:**
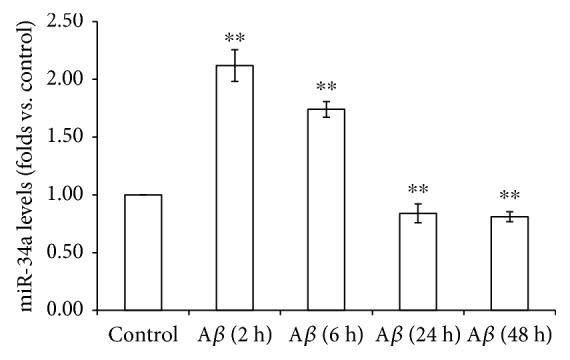
Expression of miR-34a in A*β*-treated SH-SY5Y cells. SH-SY5Y cells were exposed to A*β*1-40 (5 *μ*M) for 0, 2, 6, 24, or 48 h. Quantitative real-time PCR was used to detect miR-34a levels in different groups. The relative expression of miR-34a was normalized to the expression of the internal control. Data are presented as the means ± SEM from four independent experiments; ^∗∗^*P* < 0.01 compared with the control.

**Figure 4:**
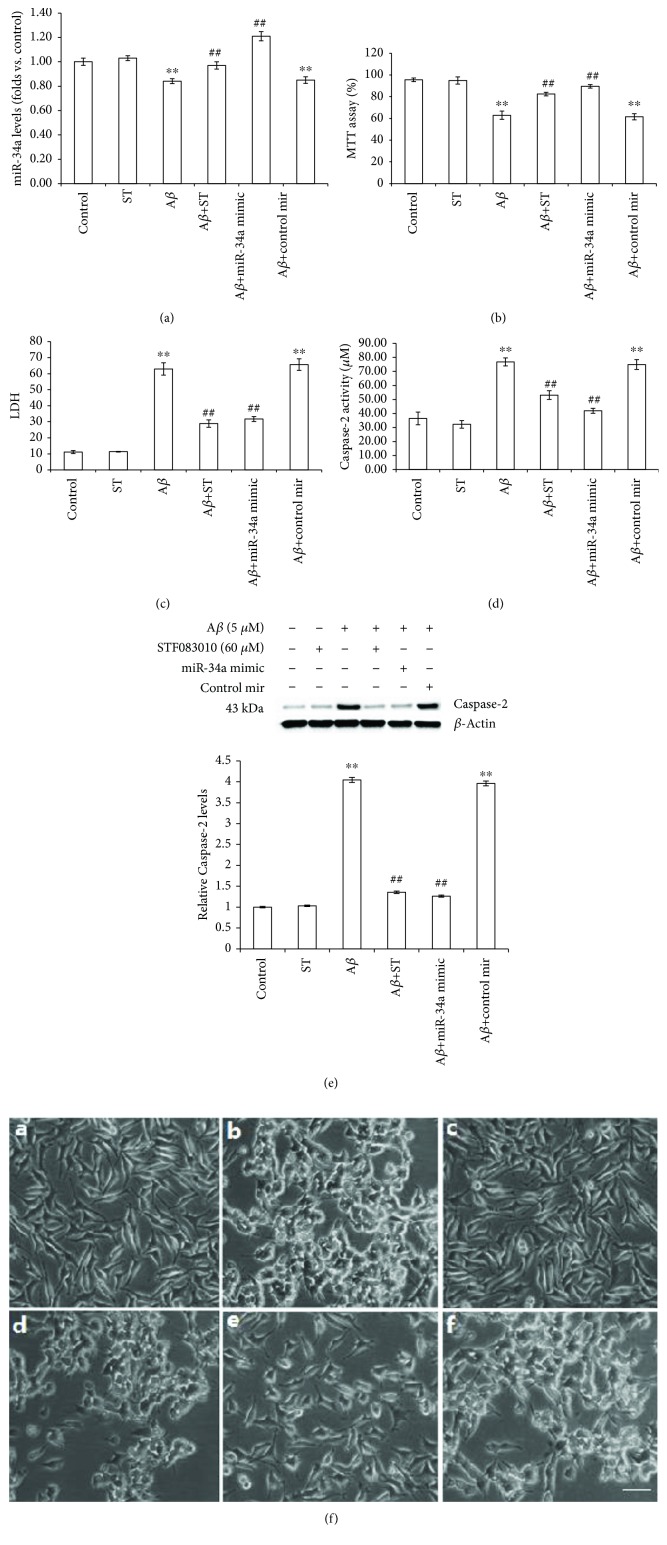
The roles of IRE1*α* and miR-34a in A*β*-induced toxicity in SH-SY5Y cells. Cells were incubated with STF-083010 (60 *μ*M)+A*β*1-40 (5 *μ*M), STF-083010 (60 *μ*M) alone, or A*β*1-40 (5 *μ*M) alone or were untreated. In transfection studies, cells were transfected with the miR-34a mimic oligonucleotide or nonspecific control in the presence or absence of A*β*1-40 (5 *μ*M). (a) miR-34a levels were measured using quantitative real-time PCR. (b) Cell viability was measured using the MTT assay. (c) The integrity of the plasma membrane was assessed by LDH release. (d) Caspase-2 activity was measured using the Caspase-2 cellular activity assay kit. (e) The expression of Caspase-2 was measured by Western blotting. (f) Morphological analysis of SH-SY5Y cells by microscopy. Scale bar: 100 mm. Data are presented as the means ± SEM from four independent experiments. ^∗∗^*P* < 0.01 compared with the control; ^##^*P* < 0.01 compared with the A*β*1-40-alone group.

To explore the mechanistic bases of IRE1*α*-mediated A*β*1-40 injury, we turned our attention to miR-34a, a candidate target of IRE1*α*'s endonuclease activity. SH-SY5Y cells were exposed to A*β*1-40 (5 *μ*M) for 0, 2, 6, or 24 h. Quantitative real-time PCR was used to determine the miR-34a levels in different groups. The level of miR-34a was increased after treatment with A*β*1-40 (5 *μ*M) for 2 h and 6 h, which peaked at 2 h and then started to decrease. At 24 h, the miR-34a levels had decreased to 84.0% of the control (Figure 3, *P* < 0.01). To confirm that IRE1*α* acts as a negative regulator of miR-34a under A*β*1-40 conditions, we examined the miR-34a levels after IRE1*α* inhibition. As shown in Figure 4(a), STF-083010 partially prevented the A*β*-induced decrease in miR-34a levels (97.0% of control levels, *P* < 0.01). Treatment with 60 *μ*M STF-083010 alone had no effect on miR-34a levels, with no significant difference compared with the control group.

### 3.4. Both IRE1*α* Inhibition and miR-34a Transfection Reduce A*β* Neurotoxicity

To determine whether IRE1*α* contributes to the A*β*-induced death of SH-SY5Y cells, we inhibited IRE1*α* using a specific inhibitor of IRE1*α*. As illustrated in Figure 4(b), A*β*1-40 (5 *μ*M for 24 h) treatment decreased cell viability to 62.93 ± 3.83%. Pretreatment with STF-083010 (60 *μ*M) significantly mitigated the A*β*1-40-induced death of SH-SY5Y cells, restoring cell viability to 82.42 ± 1.62% (*P* < 0.01). STF-083010 at 60 *μ*M alone did not cause any apparent cytotoxicity. This result was confirmed by LDH analysis, which showed that STF-083010 alleviated A*β*-induced cytotoxicity in SH-SY5Y cells. A*β*1-40 treatment increased LDH release from 11.11 ± 0.95% to 62.93 ± 3.83%. Pretreatment with STF-083010 (60 *μ*M) restored LDH release to 28.91 ± 2.30%. Taken together, these data indicate that IRE1*α* are indeed involved in A*β*-induced neurotoxicity.

We also examined cell viability in SH-SY5Y cells transfected with the miR-34a mimic oligonucleotide or a nonspecific control to determine whether miR-34a regulates cell viability in A*β*1-40-treated SH-SY5Y cells. miR-34a levels were markedly increased in SH-SY5Y cells transfected with the miRNA-34a mimic oligonucleotide (1.21-fold vs. control, *P* < 0.01, Figure 4(a)), with no change observed in SH-SY5Y cells transfected with the control oligonucleotide. As shown in Figure 4(b), the miRNA-34a mimic oligonucleotide restored cell viability to 89.53 ± 1.63% (*P* < 0.01), significantly higher than the A*β*-alone group. The cell viability in SH-SY5Y cells transfected with control oligonucleotide showed no significant changes. This result was confirmed by LDH analysis, which showed that in SH-SY5Y cells transfected with the miR-34a mimic oligonucleotide, A*β*-induced cytotoxicity was alleviated, restoring LDH release to 31.73 ± 1.51% (*P* < 0.01, Figure 4(c)).

### 3.5. Both IRE1*α* Inhibition and miR-34a Transfection Reduce the Activation of Caspase-2

We investigated Caspase-2 expression and activity after exposure to A*β*1-40 and STF-083010, as described above, to evaluate the role of IRE1*α* in A*β*-induced activation of Caspase-2 in SH-SY5Y cells. As illustrated in Figure 4(d), Caspase-2 activity was significantly increased by A*β*1-40 (2.10-fold vs. control; *P* < 0.01), which was partially prevented by STF-083010 (1.46-fold vs. control, *P* < 0.01). Treatment with 60 *μ*M STF-083010 alone had no effect on Caspase-2 activity. Western blotting demonstrated that A*β* resulted in an increase in the Caspase-2 protein expression (4.04-fold vs. control, *P* < 0.01), while Caspase-2 levels were markedly decreased in SH-SY5Y cells pretreated with STF-083010 (60 *μ*M) for 6 h (33.53% of the A*β*-alone group, Figure 4(e), *P* < 0.01).

We also examined the Caspase-2 expression and activity in SH-SY5Y cells transfected with the miR-34a mimic oligonucleotide or a nonspecific control to determine whether miR-34a regulates Caspase-2 activation in A*β*1-40-treated SH-SY5Y cells. As illustrated in Figure 4(d), after miR-34a mimic transfection, Caspase-2 activity was markedly decreased (54.58% of the A*β*-alone group, *P* < 0.01). Caspase-2 activity in SH-SY5Y cells transfected with control oligonucleotide showed no significant changes. Caspase-2 levels were markedly decreased in SH-SY5Y cells transfected with the miR-34a mimic oligonucleotide (31.2% of the A*β*-alone group, *P* < 0.01). The expression levels of Caspase-2 in SH-SY5Y cells transfected with control oligonucleotide showed no significant changes (Figure 4(e)).

### 3.6. Morphology of SH-SY5Y Cells

SH-SY5Y cells were treated according to the protocol described in Materials and Methods. As shown in Figure 4(f), visible morphological changes were observed during the treatment in cells. SH-SY5Y in the normal group adhered to the wall after 24 h of inoculation. The majority of the cells were oval and triangular in shape. The nucleus was easy to distinguish from the perikaryon. Most of the cells grew more than 2 protrusions, and most of the protrusions came into contact with adjacent cells or protrusions. However, when exposed to A*β*1-40 (5 *μ*M) for 24 h, most of the cells showed significant morphological changes, such as loss of neurites, detaching from the surface, forming clusters, and having an irregular cell shape. The number of cells injured and the degree of cell injury were significantly improved when the cells were pretreated with 60 *μ*M STF-083010 or transfected with the miR-34a mimic oligonucleotide, while STF-083010 treatment alone did not show morphological alterations, and transfection with nonspecific control oligonucleotide had no effect on improving the cell injury.

## 4. Discussion

The A*β* peptide has been reported to cause apoptosis by activating ER stress-specific caspases [5, 27]. However, induction of the IRE1 signaling pathway by A*β* was not shown in these studies. As an RNase, IRE1*α* targets and regulates the expression of a number of RNAs (both coding and noncoding) [22, 23]. As a downstream target of IRE1*α*, XBP1 is one of the most well-studied genes modulated by IRE1*α* and plays important roles in regulating cell survival and the UPR [13]. A previous study has reported that AD progression at the histopathological level is associated with chronic IRE1 activation in the brain and IRE1 deficiency significantly reduces the accumulation of A*β* in the brain of 5xFAD mice [28]. In this study, we uncovered the other role of IRE1 in the pathogenesis of AD. We investigated the effects of A*β*1-40 on activation of the IRE1 signaling pathway by determining the levels of the phosphorylated form of IRE1*α* and spliced XBP1. A*β* significantly increased the levels of the p-IRE1*α* protein and the spliced XBP1, revealing that the IRE1 signaling pathway was activated in A*β*-treated cells. The A*β* peptide induces Ca^2+^ release from the ER, potentially resulting in Ca^2+^ depletion in the ER lumen. The depletion of ER Ca^2+^ disturbs the posttranslation modification and folding of proteins, leading to the accumulation of unfolded and misfolded proteins in the ER lumen and subsequent induction of ER stress [29, 30]. The evidence provided in our study will be helpful in improving our understanding of the mechanism by which the A*β* peptide induces ER stress. The activation of the IRE1 signal pathway observed in this study may be associated with abnormal Ca^2+^ signaling.

According to recent studies, miR-34a is cleaved by IRE1*α*, resulting in activation of Caspase-2 in mouse embryonic fibroblasts (MEFs) [22, 23]. We examined the levels of miR-34a in SH-SY5Y cells treated with A*β*1-40 and STF-081030, which displays IRE1*α* RNase inhibitory activity [31], to assess whether the cleavage of miR-34a also occurs in SH-SY5Y cells treated with A*β*1-40 and investigate whether IRE1*α* is involved in this process. Our results show that IRE1 activation downregulated miR-34a expression. In previous studies, miR-34a was overexpressed in specific brain regions of patients with AD, as well as in the 3xTg-AD mouse model. However, in our study, we observed a dynamic change in miR-34a expression in A*β*1-40-treated SH-SY5Y cells. Increases and decreases in the miR-34a level were detected at different times after A*β* treatment. The discrepancies between the results from these studies and our findings indicate that the mechanism modulating the miR-34a expression differs between cell culture models and neurodegenerative conditions and another pathological process may be involved in the abnormal expression of miR-34a in the brains of patients with AD. Further studies in relevant experimental models are warranted. The present study provides the first description of miR-34a expression in response to A*β*-induced ERS, which may lead to the discovery of new mechanisms.

Based on the results from the present study, exposure to A*β* clearly resulted in a decrease in cell viability, and cell death was mediated by a caspase-dependent mechanism. Caspase-2, a member of the cysteine protease family, is one of the targets of miR-34a [22]. Our study provides evidence that downregulation of the miR-34a expression in A*β*1-40-treated SH-SY5Y cells may increase Caspase-2 activity and potentially contribute to apoptotic cell death. Additionally, an IRE1*α* inhibitor aided in preventing the effect of A*β*1-40 on miR-34a levels and Caspase-2 activity. Thus, both IRE1*α* and miR-34a are involved in A*β*-induced cytotoxicity, and this effect may be mediated by Caspase-2, a well-characterized regulator of apoptosis. Transfection of the miR-34a mimic, which protects miR-34a from degradation by IRE1, decreased the levels of the Caspase-2 protein, as shown in the Western blots. Further investigations are required to determine how Caspase-2 and its downstream targets contribute to these cellular processes during ER stress. The IRE1*α* inhibitor may have the potential to divert apoptosis in response to ER stress by modulating the miR-34a level. These results were further confirmed by morphological observation. However, we cannot conclude that miR-34a is the only microRNA involved in A*β*1-40-induced cytotoxicity, and additional analyses are necessary to explore the levels of microRNAs in clinical specimens. In addition, an examination of the protein and mRNA levels of the other known targets of miR-34a is necessary to determine whether these proteins are also involved in this process.

## 5. Conclusion

Taken together, our findings identify a previously unrecognized mechanism by which the A*β* peptide regulates Caspase-2 expression and activity to induce cytotoxicity that involves both IRE1*α* and a noncoding RNA (miR-34a). Our study may prompt the development of a novel therapeutic strategy that targets IRE1*α* and miR-34a to treat AD.

## Figures and Tables

**Table 1:** Oligonucleotide primer sets for RT-PCR.

Name	Sequence (5′–3′)	Length	Tm	Size
XBP1 F	TTACGAGAGAAAACTCATGGCC	22	58.9	283
XBP1 R	GGGTCCAAGTTGTCCAGAATGC	22	63.1	
*β*-Actin F	CTTAGTTGCGTTACACCCTTTCTTG	25	62	156
*β*-Actin R	CTGTCACCTTCACCGTTCCAGTTT	24	64.4	

**Table 2:** Oligonucleotide primer sets for real-time PCR.

Name	Sequence (5′–3′)	Length	Tm	Size
miR-34a F	GATCGATGGCAGTGTCTTAGCT	22	58.7	62
miR-34a R	GTGCAGGGTCCGAGGTATTC	20	59.2	
U6 F	CTCGCTTCGGCAGCACA	17	60.4	94
U6 R	AACGCTTCACGAATTTGCGT	20	59.7	

## Data Availability

The data used to support the findings of this study are available from the corresponding author upon request.

## References

[1] Almkvist O. (2000). Functional brain imaging as a looking-glass into the degraded brain: reviewing evidence from Alzheimer disease in relation to normal aging. *Acta Psychologica*.

[2] Annaert W., De Strooper B. (2002). A cell biological perspective on Alzheimer’s disease. *Annual Review of Cell and Developmental Biology*.

[3] Selkoe D. J. (2004). Cell biology of protein misfolding: the examples of Alzheimer’s and Parkinson’s diseases. *Nature Cell Biology*.

[4] Taylor J. P., Hardy J., Fischbeck K. H. (2002). Toxic proteins in neurodegenerative disease. *Science*.

[5] Nakagawa T., Zhu H., Morishima N. (2000). Caspase-12 mediates endoplasmic-reticulum-specific apoptosis and cytotoxicity by amyloid-beta. *Nature*.

[6] Boyce M., Yuan J. (2006). Cellular response to endoplasmic reticulum stress: a matter of life or death. *Cell Death and Differentiation*.

[7] Paschen W., Mengesdorf T. (2005). Endoplasmic reticulum stress response and neurodegeneration. *Cell Calcium*.

[8] Chen Y., Brandizzi F. (2013). IRE1: ER stress sensor and cell fate executor. *Trends in Cell Biology*.

[9] Han D., Lerner A. G., Vande Walle L. (2009). IRE1*α* kinase activation modes control alternate endoribonuclease outputs to determine divergent cell fates. *Cell*.

[10] Plongthongkum N., Kullawong N., Panyim S., Tirasophon W. (2007). Ire1 regulated XBP1 mRNA splicing is essential for the unfolded protein response (UPR) in Drosophila melanogaster. *Biochemical and Biophysical Research Communications*.

[11] Tam A. B., Koong A. C., Niwa M. (2014). Ire1 has distinct catalytic mechanisms for XBP1/HAC1 splicing and RIDD. *Cell Reports*.

[12] Uemura A., Oku M., Mori K., Yoshida H. (2009). Unconventional splicing of XBP1 mRNA occurs in the cytoplasm during the mammalian unfolded protein response. *Journal of Cell Science*.

[13] Wu R., Zhang Q. H., Lu Y. J., Ren K., Yi G. H. (2015). Involvement of the IRE1*α*-XBP1 pathway and XBP1s-dependent transcriptional reprogramming in metabolic diseases. *DNA and Cell Biology*.

[14] Hetz C., Martinon F., Rodriguez D., Glimcher L. H. (2011). The unfolded protein response: integrating stress signals through the stress sensor IRE1*α*. *Physiological Reviews*.

[15] Hollien J., Weissman J. S. (2017). Decay of endoplasmic reticulum-localized mRNAs during the unfolded protein response. *Science*.

[16] Sonkoly E., Pivarcsi A. (2009). MicroRNAs in inflammation. *International Reviews of Immunology*.

[17] van Rooij E., Olson E. N. (2012). MicroRNA therapeutics for cardiovascular disease: opportunities and obstacles. *Nature reviews Drug Discovery*.

[18] Lin S., Gregory R. I. (2015). MicroRNA biogenesis pathways in cancer. *Nature Reviews Cancer*.

[19] Wong M. Y., Yu Y., Walsh W. R., Yang J. L. (2011). MicroRNA-34 family and treatment of cancers with mutant or wild-type p53 (review). *International Journal of Oncology*.

[20] Qi Y. T., Zhao Y., Zhang Z. (2009). The expression pattern and possible function of microRNA-34a in neurons. *Journal of Molecular Cell Biology*.

[21] Wang X., Liu P., Zhu H. (2009). miR-34a, a microRNA up-regulated in a double transgenic mouse model of Alzheimer’s disease, inhibits bcl2 translation. *Brain Research Bulletin*.

[22] Upton J.-P., Wang L., Han D. (2012). IRE1*α* cleaves select microRNAs during ER stress to derepress translation of proapoptotic caspase-2. *Science*.

[23] Hassler J., Cao S. S., Kaufman R. J. (2012). IRE1, a double-edged sword in pre-miRNA slicing and cell death. *Developmental Cell*.

[24] Li Q., Fang J., Yang M., Wu D., Zhang L., Zhang Y. (2010). Galantamine inhibits calpain-calcineurin signaling activated by beta-amyloid in human neuroblastoma SH-SY5Y cells. *Neuroscience Letters*.

[25] Papandreou I., Denko N. C., Olson M. (2011). Identification of an IRE1*α* endonuclease specific inhibitor with cytotoxic activity against human multiple myeloma. *Blood*.

[26] Livak K. J., Schmittgen T. D. (2001). Analysis of relative gene expression data using real-time quantitative PCR and the 2^−ΔΔC^_T_ method. *Methods*.

[27] Hitomi J., Katayama T., Eguchi Y. (2004). Involvement of caspase-4 in endoplasmic reticulum stress-induced apoptosis and A*β*-induced cell death. *The Journal of Cell Biology*.

[28] Duran-Aniotz C., Cornejo V. H., Espinoza S. (2017). IRE1 signaling exacerbates Alzheimer’s disease pathogenesis. *Acta Neuropathologica*.

[29] Mattson M. P., LaFerla F. M., Chan S. L., Leissring M. A., Shepel P. N., Geiger J. D. (2000). Calcium signaling in the ER: its role in neuronal plasticity and neurodegenerative disorders. *Trends in Neurosciences*.

[30] Paschen W. (2000). Role of calcium in neuronal cell injury: which subcellular compartment is involved?. *Brain Research Bulletin*.

[31] Kriss C. L., Pinilla-Ibarz J. A., Mailloux A. W. (2012). Overexpression of TCL1 activates the endoplasmic reticulum stress response: a novel mechanism of leukemic progression in mice. *Blood*.

